# NSF NeXUS: A New Model for Accessing the Frontiers of Ultrafast Science

**DOI:** 10.1021/acscentsci.4c01682

**Published:** 2025-01-06

**Authors:** L. Robert Baker, Louis F. DiMauro, Claudia Turro, Jay A. Gupta, Roland K. Kawakami, Thomas K. Allison, Theodore J. Ronningen, Timothy D. Scarborough, Vyacheslav Leshchenko, Seth S. Shields, John E. Beetar

**Affiliations:** † Chemistry and Biochemistry, The Ohio State University, 100 W 18th Ave, Columbus, Ohio 43210, United States; ‡ Physics, The Ohio State University, 191 W Woodruff Ave, Columbus, Ohio 43210, United States; § Chemistry, Physics and Astronomy, Stony Brook University, Stony Brook, New York 11794, United States; ∥ Electrical and Computer Engineering, The Ohio State University, 2015 Neil Ave, Columbus, Ohio 43210, United States; ⊥ NeXUS, The Ohio State University, 120 W 18th Ave, Columbus, Ohio 43210, United States

## Abstract

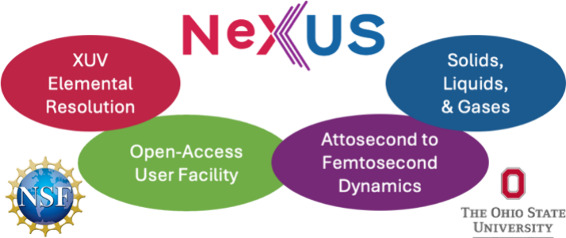

NSF NeXUS is an open-access user facility that enables observation of electron motion with sub-femtosecond time resolution, angstrom spatial resolution, and element-specific spectral resolution.

The coupled dynamics of electrons and nuclei are the primary process underlying important applications ranging from solar energy conversion to information storage and processing. Treatment of electronic motion and nuclear dynamics as decoupled is the fundamental assumption of the Born–Oppenheimer approximation, which enables a quantum mechanical description of many chemical systems. However, in reality, numerous processes proceed via strongly coupled electron and nuclear dynamics where photoexcitation,^1−3^ electrical bias,^4,5^ magnetic switching,^6−8^ and nonadiabatic energy dissipation at conical intersections,^9,10^ all play key roles in guiding the flow of energy in molecules and materials. The National eXtreme Ultrafast Science (NeXUS) facility was designed and constructed to enable direct observation of electronic and nuclear motion with attosecond to femtosecond time resolution, angstrom spatial resolution, and element-specific spectral resolution.

The scientific goal to understand and control reaction dynamics in molecular and material systems on the fundamental time scale of electron motion is inherently an interdisciplinary pursuit bridging many fields of science and engineering. In the Bohr model of the atom, it takes approximately 150 attoseconds for an electron to orbit the hydrogen atom, and in complex systems, attosecond electron dynamics determine material properties. For example, in strongly correlated materials, electron interactions drive emergent quantum behaviors such as ultrafast metal-to-insulator phase transitions^11,12^ or the formation of transient topological defects.^13,14^ Chemical reactions proceed via the formation and cleavage of chemical bonds, which occur on the time scale of molecular vibrations;^15,16^ however, these nuclear dynamics are often set in motion by even faster electronic transitions.^17−19^ In biological systems, light harvesting complexes rely on efficient energy transfer, which may occur via a combination of electronic and nuclear coherence.^20−23^ Understanding these ultrafast dynamics can advance multiple fields, including photochemistry,^19^ electrochemistry,^24,25^ catalysis,^26^ spin crossover,^27−29^ and magnetization switching.^6−8^ In addition, controlling ultrafast processes is key to developing new technologies to address pressing questions related to energy conversion from renewable sources, green chemical synthesis, mimicking biological functions, and creating new platforms for information storage and processing.

With this in mind, it is not surprising that multiple national studies and community reports have emphasized the need to understand and control energy transport and chemical reactions on the scale of individual electrons and atoms.^30−34^ For example, the US National Science Foundation (NSF) highlighted the ability to “observe, manipulate, and control the behavior of particles and energy on atomic and sub-atomic scales” as one of the 10 Big Ideas that will shape the future.^33^ Similarly, the US Department of Energy Basic Energy Sciences Advisory Committee, in their report “Five Challenges for Science and the Imagination”, posed the question, “How do we control material processes at the level of electrons?”.^30^ Although widely acknowledged as a grand challenge, this goal can never be realized without the ability to directly observe the underlying electronic and nuclear dynamics on the relevant scales of time and space. This calls for putting techniques capable of attosecond to femtosecond time resolution, angstrom spatial resolution, and element-specific spectral resolution directly into the hands of the scientific community.

Due to the need for short pulses of light to probe fast processes, ultrafast science has historically followed the development of short pulse lasers. The development and rapid commercialization of femtosecond laser technology in the early ’90s enabled ultrafast studies of chemical reactions and were recognized by the 1999 Nobel Prize in Chemistry to Ahmed Zewail.^16,35^ However, the shortest laser pulses available from Ti:sapphire lasers have saturated at approximately 10 fs with few cycle pulses possible using nonlinear pulse compression. At 800 nm central wavelength, a single optical cycle is approximately 2.4 fs, indicating that higher frequencies are required to reach attosecond pulse durations. An alternative platform for short pulse generation is based on laser-driven high harmonic generation (HHG), which produces broadband pulses of extreme ultraviolet (XUV) and soft X-ray frequencies.^36−38^ HHG is an inherently attosecond process that produces XUV light with sub-femtosecond durations based on strong field rescattering physics.^39−42^ Recognizing the enormous potential of attosecond XUV light sources to study dynamics on previously inaccessible time scales, this work was recognized by the 2023 Nobel Prize in Physics to Pierre Agostini, Anne L’Huillier, and Ferenc Krausz.

Even for processes that do not require attosecond time resolution, an HHG light source offers a number of advantages where XUV light provides an effective probe of ultrafast dynamics not accessible at visible wavelengths.^43,44^ In addition to unprecedented time resolution, XUV and soft X-ray spectroscopies measure element-specific, core-to-valence electronic transitions. This is illustrated in Figure 1, which shows element-specific absorption spectra at the M_2,3_-edge of a series of early to late 3d transition metals measured using a tabletop HHG light source.^45^ The shape of these absorption edges provides detailed information including the oxidation state, spin state, and ligand coordination of the respective metal centers.^46−48^ This means that in time-resolved X-ray measurements, it is possible to resolve the contribution of individual atoms to the overall charge and spin dynamics of a material, enabling a real-time understanding of electron motion in important chemical systems. For example, CuFeO_2_ is a mixed-valence metal oxide with promise for conversion of CO_2_ to fuels using sunlight. The activity and selectivity of this material are greatly enhanced relative to monometallic Fe or Cu oxides alone, suggesting that an electronic interaction between Cu and Fe metal centers facilitates photocatalytic activity. Using ultrafast XUV spectroscopy, it was recently shown that the formation of a Cu → Fe metal-to-metal charge transfer state leads to spatial charge separation in this material on the femtosecond time scale.^49^ This finding was enabled by simultaneous measurements at the Fe M_2,3_-edge, Cu M_2,3_-edge, and O L_1_-edge of this material, illustrating how element-specific measurements provide detailed information that is nearly impossible to obtain by ultrafast optical spectroscopy lacking element-specific resolution. Similar benefits of XUV spectroscopy have been demonstrated for a variety of other molecular and material systems, including the study of conical intersections during photodissociation and ring opening reactions,^50,51^ energy relaxation pathways in photocatalytic complexes,^52−54^ and phase transitions and magnetization dynamics in condensed matter systems.^55−57^

**Figure 1:**
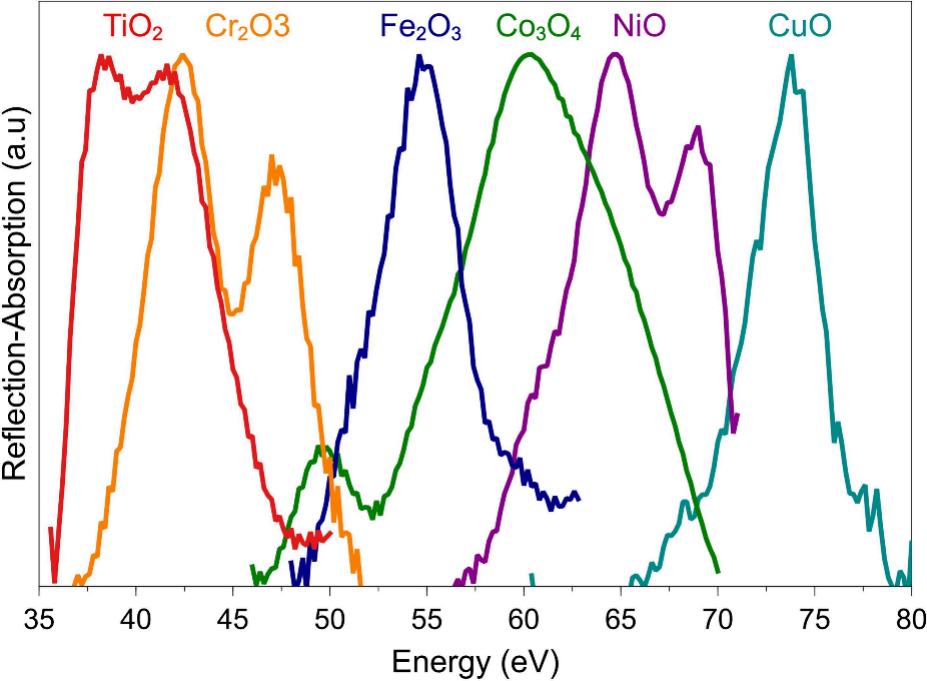
Element-specific absorption spectra at the M_2,3_-edge of a series of early to late 3d transition metals measured using a tabletop HHG light source. Reproduced from ref (45). Copyright 2022 American Chemical Society.

In addition to traditional XUV absorption spectroscopy, coupling XUV light with scanning tunneling microscopy (STM) extends the capabilities of STM by using core-to-valence excitation in the tip–sample junction to provide element-specific contrast to atomic-resolution imaging. Beyond the element specificity of XUV absorption, ultrafast XUV pulses offer additional advantages for probing dynamics in condensed phase systems, including excitonic, ferroelectric, topological, superconducting, and valley-specific phenomena. For example, photoemission spectroscopy with XUV light samples the entire Brillouin zone, something that is not possible using visible or UV sources. For time-resolved photoemission, high repetition rates are needed to minimize effects of sample space charge, and narrow bandwidths are required to maintain high electron energy resolution. Consequently, XUV light sources with durations from sub-femtosecond to hundreds of femtoseconds, repetition rates from 100 kHz to multiple MHz, and bandwidths from tens of eV to meV are needed to support a wide range of scientific applications.

Despite the many benefits of ultrafast spectroscopy using XUV light, the development of HHG light sources has proliferated more slowly compared to femtosecond laser technology. The reasons for this are two-fold. First, HHG is a relatively inefficient process resulting in a much lower flux of XUV light compared to amplified, femtosecond lasers. Given an approximate conversion efficiency of 10^–6^–10^–8^, XUV light produced by HHG is typically limited to small (nJ) pulse energies. Effectively scaling the HHG process to produce higher flux requires increasing the repetition rate by using ultrafast lasers with high average power, and kilowatt-class ultrafast lasers are only recently becoming accessible. The second reason for slow proliferation of this technology is that the physical instrumentation required to construct an HHG generation source, beamline, and end station is complex and expensive, making widespread adoption and commercialization of the technology challenging. For these reasons, ultrafast XUV spectroscopy has remained largely confined to a relatively small group of scientists with expertise in ultrafast optics, indicating that the full impact of these capabilities on multiple scientific disciplines is yet to be realized. Early career researchers and scientists from primarily undergraduate and/or minority serving institutions are especially affected by limited access to these enabling technologies.

The benefits of ultrafast XUV light and the challenges of the technology motivate the effort to implement creative platforms to democratize the research enterprise by putting these cutting-edge tools into the hands of a scientifically diverse user community. The need to make these tools widely accessible was highlighted in a 2018 report by the National Academy of Science titled “Reaching for the Brightest Light: Opportunities in Intense Ultrafast Lasers”.^58^ This report advocates for investment in research capabilities uniquely enabled by high-intensity, ultrafast laser light to facilitate a quantum leap in ultrafast science and technology. Efforts to expand the scientific impact of these light sources are moving forward in Europe through efforts such as the Extreme Light Infrastructure (ELI) and Laserlab Europe. Similar efforts have followed in the United States, such as LaserNetUS, the Brightest Light Initiative, and now several NSF Mid-Scale Research Infrastructure (MSRI) facilities, including NeXUS.

User facilities are demonstrated solutions to overcoming technological research barriers, and attosecond XUV technologies are particularly well-matched to a mid-scale user facility. A user facility focused on ultrafast dynamics can nimbly support a broad cross section of science spanning disciplines such as atomic, molecular, and optical physics, chemical dynamics, surfaces science and catalysis, condensed matter physics, and ultrafast processes in biology. Excitingly, given the tabletop nature of this technology, it is significantly less expensive to implement and maintain than large-scale facilities, such as a synchrotron or X-ray free electron laser (XFEL). The NSF identified the impact a mid-scale facility could have on the researcher community by naming mid-scale infrastructure as one of the 10 Big Ideas predicted to shape the future of US science.^33^ In the first NSF call for MSRI facilities, the NSF selected NeXUS as one of ten facilities that could meet critical needs of the scientific community. The Ohio State University agreed to host NeXUS, and NSF funded its development. The mission of NeXUS fits the goals originally envisioned under the MSRI program by providing broad community access by scientifically diverse users to state-of-the-art capabilities enabled by ultrafast XUV light. NeXUS design and construction began in 2019, and now NeXUS will be accessible to the community through an open access process.

At the heart of the NeXUS system is an ultrafast, kilowatt-class laser that produces femtosecond pulses of near-infrared light to drive HHG at a 100 kHz to >5 MHz repetition rate. This laser, which was acquired from Active Fiber Systems, provides nearly 2 orders of magnitude higher average power than traditional Ti:sapphire systems. A large pulse energy (1–8 mJ) for generating XUV light at a high repetition rate is realized by coherently combining the output of 16 Yb-doped fiber amplifiers. This scalable approach provides more than 800 W of average power at 240 fs pulse duration. Two stages of nonlinear pulse compression reduce the pulse duration to 35 fs at 550 W average power and 8 fs at 250 W average power, respectively. These pulses are then used to produce XUV, with design plans for soft X-ray light, that is delivered to a suite of beamlines and characterization end stations, supporting a breadth of cross-cutting science. By enabling HHG at these high repetition rates, the mid-scale NeXUS facility is bridging the gap between traditional tabletop HHG light sources and large-scale facilities, such as XFELs, as shown in Figure 2.

**Figure 2:**
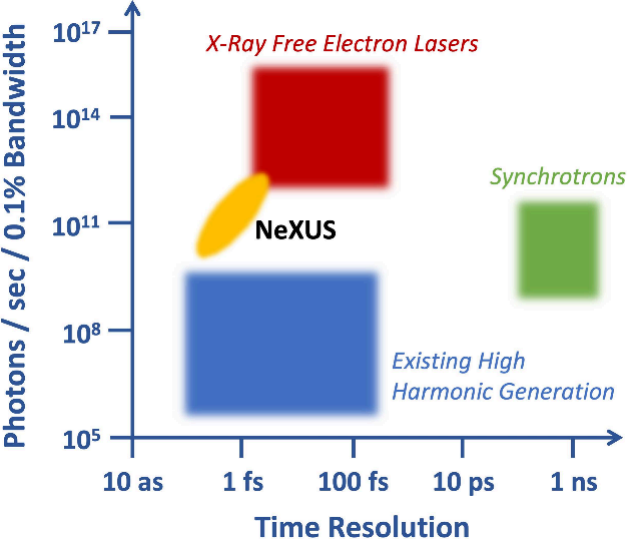
Comparison of the range of photon flux and time resolution available from X-ray sources.

The combination of attosecond pulses, XUV and soft X-ray photon energies, and high repetition rate coupled with various end stations for molecular and material characterization will enable measurements at NeXUS that have previously been difficult to access. Figure 3 shows a diagram of the NeXUS facility where laser light is generated, compressed, and directed into one of three beamlines to produce XUV light, which is then coupled to the various experimental end stations. In each of the end stations, tunable pump pulses are available to excite the sample, and XUV light is available to probe the resulting ultrafast dynamics. Photon budgets, spectral range, bandwidths, and pulse durations available in each end station are shown in Figure 4. NeXUS will specifically support the following experiments:

- **Time-Resolved X-ray Absorption/Reflection Spectroscopy (TR-XAS/XRS)**—A combination of end stations enables XUV and soft X-ray absorption or reflection spectroscopy of molecules and materials in the solid, liquid, or gas phase with attosecond to femtosecond time resolution. These experiments measure the excited state spectrum after interaction of the sample with a tunable wavelength pump pulse. End stations are available to support the following experiments:
  - *Transmission or Variable-Angle Reflection from Solids*—This end station provides the ability to position solid samples (e.g., thin films, epitaxial layers, or single crystals) for measuring X-ray transmission or variable-angle reflection. Samples are raster scanned during analysis to avoid material damage.
  - *Liquid Sheet*–The ability to generate thin liquid sheets with a thickness below 100 nm enables ultrafast XUV transient absorption studies of liquids and solvated molecules.
  - *XUV Magnetic Circular Dichroism—*A broadband circular polarizer coupled with sample magnetization enables time-resolved XUV magnetic circular dichroism (XMCD) studies.

**Figure 3:**
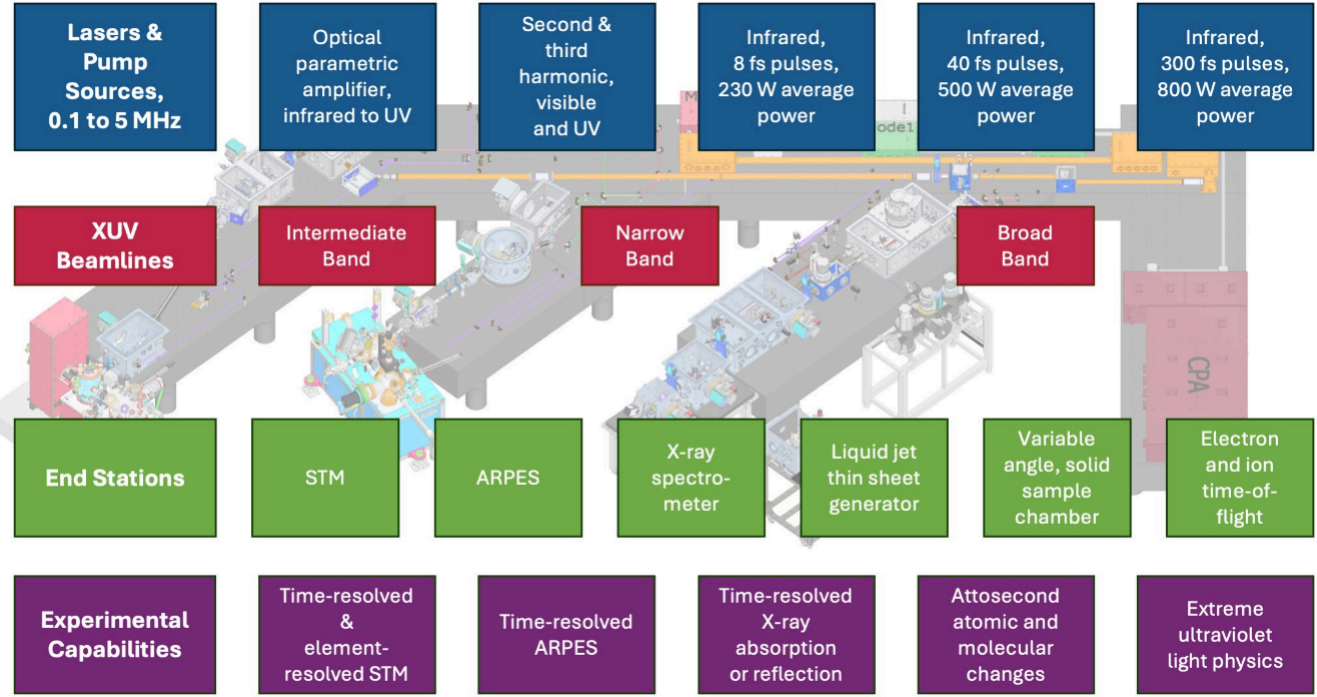
Laboratory layout, key components, and key capabilities of the NeXUS system.

**Figure 4:**
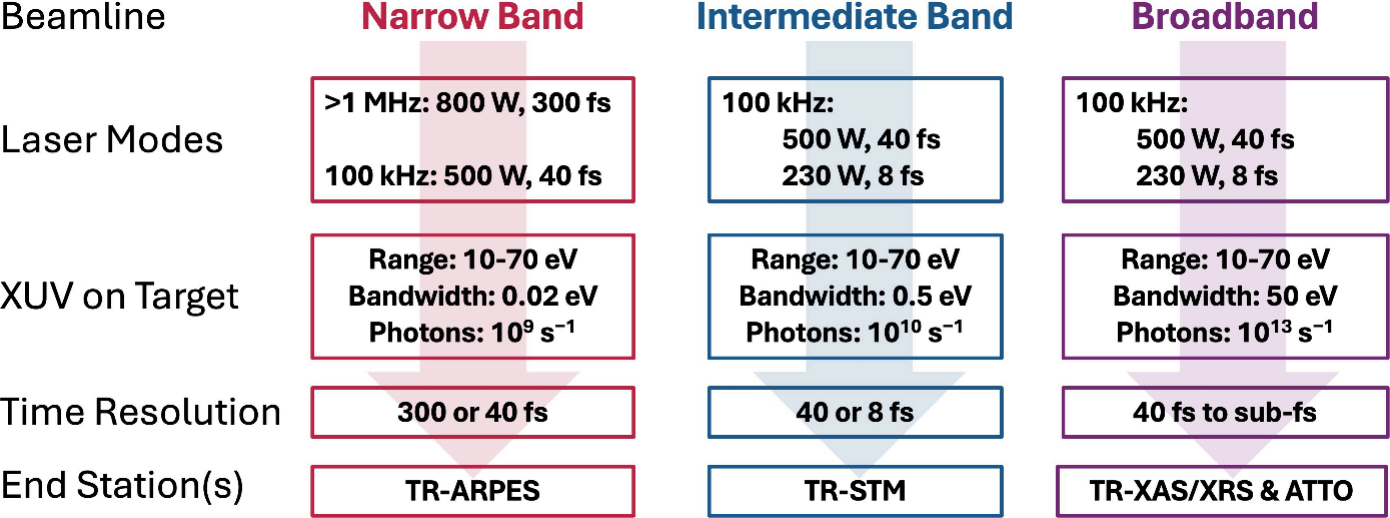
Initial use specifications for the NeXUS beamlines. For the intermediate and broadband beamlines, NeXUS plans to extend the XUV energy range into soft X-rays in the next two years, reaching at least the carbon K-edge at 284 eV.

Anticipated user experiments will provide understanding of charge and spin transport during solar energy conversion, heterogeneous catalysis, and spin transitions in molecular and extended magnets.

- **Time-Resolved Scanning Tunneling Microscopy (TR-STM)**—This system couples tunable, monochromatic, XUV, and soft X-ray pulses to a scanning tunneling microscope (STM) to enable element-specific contrast with ultrafast time resolution and atomic-scale spatial resolution. This will enable studies of temporally and spatially resolved charge and spin dynamics of quantum point defects and surface chemical reactions.
- **Time-Resolved and Angle-Resolved Photoemission Spectroscopy (TR-ARPES)**—This system enables time and angle-resolved photoemission measurements to investigate dynamic processes in molecules and materials including electron relaxation, exciton dynamics, evolution of spin-momentum locked states, and dynamics of correlated states. High data rates are obtained at this end station using the combination of MHz HHG pulses and a state-of-the-art momentum microscope photoelectron analyzer.
- **Time-Resolved Laser-Induced Electron Diffraction and Attosecond Science (TR-LIED/ATTO)**—This system can time-resolve the angular momentum distribution of gas-phase molecules probed by an intense mid-infrared pulse. The result is the ability to create movies of molecular dynamics with sub-femtosecond time resolution and sub-angstrom spatial resolution.

As described above, NeXUS is designed to support researchers from a wide range of scientific disciplines. The full value of NeXUS will be realized through its users and their experiments, and the NeXUS organizational structure is designed to maximize the impact of this significant investment. At the heart of the NeXUS facility is a team of highly qualified scientists, technicians, and administrators who support user experiments (at all stages), maintain the facility to ensure it meets user needs, and continuously improve the facility to grow its capacity and capabilities over time. Importantly, system scientists (like beamline scientists at synchrotrons) will provide technical expertise needed to support users to plan and execute experiments. NeXUS staff will also support users with proposal submission, safety training, site and data access, and other logistics necessary to execute a successful experimental campaign. Because the facility staff and equipment maintenance costs are provided by the NSF, access to the facility is free of charge for fundamental scientific research.

Access to NeXUS capabilities is administered by a peer review process based on scientific merit and broader impacts of the proposed experiments. Owing to the complexity of this state-of-the-art facility, each of the experimental capabilities will become accessible through a user-assisted commissioning process that will occur in stages during the first few years of facility operation. The NeXUS facility will issue two calls per year for user proposals, and these calls, which focus on specific experiments, will be open to all researchers regardless of institutional affiliation or scientific discipline. NeXUS will host an annual user meeting to share the scientific accomplishments of users and to support new potential users in the planning and preparation of experiments. The goal is to utilize the unique capabilities of the NeXUS facility to democratize the ultrafast scientific enterprise and make these cutting-edge resources widely accessible to the broader user community.

Directing charge and energy transfer on the scale of individual electrons and atoms requires the ability to probe dynamics on the fundamental time scales of electronic and nuclear motion. This is a goal that spans chemistry, physics, materials science, engineering, and biology. Because NeXUS was first envisioned and subsequently created to meet the identified needs of these research communities, the success of this endeavor will now depend on broad community participation. Working together, NeXUS, NSF, and facility users now can shape the future of ultrafast science.
